# Recent Trends in Biosensing and Diagnostic Methods for Novel Cancer Biomarkers

**DOI:** 10.3390/bios13030398

**Published:** 2023-03-18

**Authors:** Jagadeeswara Rao Bommi, Shekher Kummari, Kavitha Lakavath, Reshmi A. Sukumaran, Lakshmi R. Panicker, Jean Louis Marty, Kotagiri Yugender Goud

**Affiliations:** 1 School of Medicine, Case Western Reserve University, Cleveland, OH 44106, USA; 2 Department of Chemistry, Indian Institute of Technology Palakkad, Palakkad 678 557, Kerala, India; 3 Université de Perpignan Via Domitia, 52 Avenue Paul Alduy, 66860 Perpignan, France

**Keywords:** cancer diagnosis, biomarkers, electrochemical biosensors, optical biosensors, aptamers, antibodies, hybrid nanocomposites, recognition elements

## Abstract

Cancer is one of the major public health issues in the world. It has become the second leading cause of death, with approximately 75% of cancer deaths transpiring in low- or middle-income countries. It causes a heavy global economic cost estimated at more than a trillion dollars per year. The most common cancers are breast, colon, rectum, prostate, and lung cancers. Many of these cancers can be treated effectively and cured if detected at the primary stage. Nowadays, around 50% of cancers are detected at late stages, leading to serious health complications and death. Early diagnosis of cancer diseases substantially increases the efficient treatment and high chances of survival. Biosensors are one of the potential screening methodologies useful in the early screening of cancer biomarkers. This review summarizes the recent findings about novel cancer biomarkers and their advantages over traditional biomarkers, and novel biosensing and diagnostic methods for them; thus, this review may be helpful in the early recognition and monitoring of treatment response of various human cancers.

## 1. Introduction

### 1.1. Cancer

One of the major life-threatening health issues in this world is cancer. It has become the second leading cause of death, around 75% of cancer deaths transpiring in low- to middle-income countries. It causes a heavy global economic cost estimated at more than trillion dollars per year [1,2,3]. In the course of cancer, cells can grow uncontrollably as well as expand to other parts of the organs in body. Cancer can occur from the transformation of normal cells into tumor cells. Tumor cells are classified as benign and malignant. Tumors that stick to their primary location without occupying distant parts of the body are called benign tumors; these are likely to grow slowly and are not problematic. Fibroids in the uterus are an example of benign tumors. Some of the benign tumors can change into malignant tumors (e.g., colon polyps); these can be removed surgically. Tumor cells that can grow uncontrollably and spread from their primary location to distant sites are called malignant tumors; these are cancerous (i.e., invade from other parts). Malignant tumors can rapidly spread to distant parts through blood or lymph stream; this process is called as metastasis. Omnipresent cancers are the primary reason of death in the patients with cancer. Most frequently metastasis can be found in brain, lungs, liver and bone [4].

Cancers can be grouped into different categories. Mostly, the group that the cancer belongs to is determined based on the type of cells or tissues it is producing. The following are some of the following important groups in this category. 

Carcinoma: Most common type of cancer. It starts in epithelium, which is the tissue that lines or covers internal organs and passageways in the human body as well as skin. It appears in the form of tumors, which can form on lungs, breasts, skin, pancreas, kidneys, prostate, colon, etc. There are various subtypes, including adeno-carcinoma, SCC (squamous cell carcinoma), ductal carcinoma, TCC (transitional cell carcinoma) and (BCC) basal cell carcinoma. 

Sarcoma: This is a type of cancer that arises in connective tissue and/or supportive tissue such as muscle, cartilage, bone, blood vessels or fat.

Leukemia: This type is also known as blood cancer/cancer of WBC (white blood cells). It can appear in tissues which can produce blood cells (e.g., bone marrow).

Myeloma and Lymphoma: This type of cancer occurs in the cells present in the immune system (myeloma: Starts in plasma cells present inside bone marrow; lymphoma: Starts in lymphatic system such as spleen, lymph nodes).

Spinal cord and brain cancers: These cancers occur in spinal cord and brain, and are also known as central nervous system cancers.

**Figure 1:**
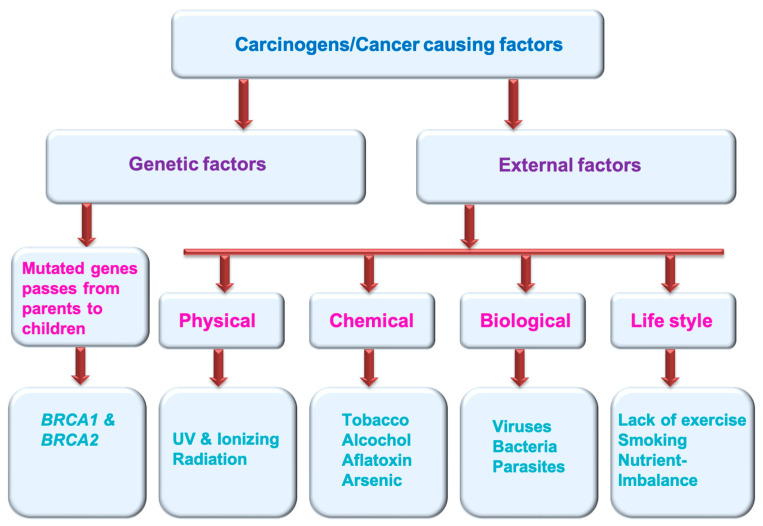
Cancer causing factors/carcinogens.

Multiple factors can generate cancer. These cancer-causing agents are termed carcinogens (Figure 1). These could be genetic factors (mutated genes pass from parents to children and cause various cancers, e.g., *BRCA1* and *BRCA2*) and external factors (physical carcinogens: Ionizing and UV radiation; tobacco smoke, alcohol, aflatoxin and arsenic are the examples of chemical carcinogens; certain viruses, bacteria, fungus and some type parasites are considered biological carcinogens; life style factors: lack of exercise, smoking, nutrient imbalance) [5] (https://www.who.int/news-room/fact-sheets/detail/cancer) (accessed on 11 November 2022). 

### 1.2. Importance of Cancer Diagnostics 

Early diagnosis of cancer has held great assurance and intuitive interest in the medical community for over 100 years [6,7]. However, delayed identification and imperfect prognosis are major causes for very low survival rate in various patients with cancer [8]. Many cancers can be treated effectively and cured if detected at the primary stage. Patients diagnosed with cancer at earlier stages are not only likely to survive, but, importantly, they also experience better care, minimal treatment morbidity, and enhanced quality of life when compared with patients with late diagnosis [9,10]. Improving earlier detection of cancers is a complex and multifaceted process. Few recent patient behaviors could be helpful for the earlier diagnosis of various cancers. One is attending cancer screening procedures, which aim to identify the cancer in asymptomatic condition (e.g., mammography to detect breast cancer), and the other is promptly introducing the patient with any potential cancer symptoms to primary care providers [11]. The necessity of symptomatic presentation to primary care is emphasized by improving public perception of the early indications of cancer. In recent years, cancer awareness is also increasing in developing and lesser-income countries. Raising public awareness, promoting visits to a healthcare provider, and diagnosing a patient at an earlier stage of the cancer can provide the opportunity to offer better treatment [12,13,14]. Nowadays, around 50% of cancers are detected at late stages, leading to serious health complications and death [12]. Early diagnosis of cancer significantly increases the efficient treatment and high chances of survival. 

### 1.3. Traditional Screening Techniques 

Cancer screening techniques have significantly promoted the decline of morbidity as well as mortality of cancer. Methods to enhance the choice of candidates for cancer screening, to acknowledge the biological foundation of cancer formation and development of novel technologies for the tumor screening would allow for advancement in the tumor screening process over time [15,16]. Screening in healthy as well as high-risk populations provides the chance to detect cancer at an early stage and with an expanded chance for treatment. Nowadays, screening techniques play a crucial role in detecting specific cancer types. However, each screening technique has some limitations, and upgraded screening techniques are very much essential to identify cancer early in healthy populations [17,18,19]. Identifying tumors at their primary stage often delivers the finest probability for a cure, which is why it is always crucial for the general population to talk with their health care providers about the types of screening that might be required. Research studies explain that early screening process can save many lives by identifying cancer in primary stages. Various medical communities and patient advocacy groups have drafted guidelines for cancer screening. In general, primary health care providers use the following approaches for tumor diagnosis [20,21,22]. 

Physical exam: During this exam, a provider can perform a scan to detect any abnormalities in body, such as skin color change, formation of lumps or abnormal growth of a tissue or an organ. This may provide the indication of the tumor.

Laboratory tests: Some lab procedures such as blood and/or urine tests can help to detect any cancer-related anomalies in body. For example, simple blood work called complete blood cell count might disclose any abnormal number or type of WBC (white blood cells) in leukemia patients.

Genetic tests: These are also lab-based tests. In these procedures, cells or tissues are examined to observe any modifications in their genes and/or chromosomes. Any of those differences might be an indication that a person is at risk of encountering a particular problem or condition.

Imaging tests: Imaging tests can examine bones or any interior organs through noninvasive method. Common cancer diagnosing imaging methods are a CT (computerized tomography) scan, X-ray, PET (positron emission tomography) scan, ultrasound, and MRI (magnetic resonance imaging) [23,24].

Biopsy: During this procedure, samples of cells are collected for examining in the laboratory. Collection of a sample can be performed by a variety of methods. The suitable type of biopsy is based on the cancer type and cancer location in body. In many cases, biopsy is the exclusive method for conclusive cancer diagnosis [25,26,27].

#### 1.3.1. Advantages of Traditional Cancer Screening

1. It can help to detect the cancer before it spreads, when it is easier to treat.
2. It can provide an advantage of early detection, which might lead to lesser recovery time and no intense treatment.
3. It can provide a better chance of survival.
4. It offers flexibility to start early treatment before symptoms appear.
5. It can also reassure a person if the screening result is normal [28,29,30].

#### 1.3.2. Limitations of Traditional Cancer Screening

1. Sometimes a false-positive test result suggests a cancer-positive status, even though no cancer is present.
2. Sometimes false-negative test results may not detect cancer, even though it is present.
3. Some screening tests might lead to more detection tests and procedures that can be painful.
4. Over-diagnosis causes needless anxiety.
5. Some screenings might cause potential issues (e.g., colon cancer screening may cause tear in colon lining).
6. These screening methods are high cost.
7. Test availability is limited to metro cities only [28,29,30].

There is a strong necessity to develop rapid and affordable screening methods for cancer diagnosis. Cancer biomarker screening through clinical and point-of-care diagnostic methods is a promising tool for an early diagnosis of cancer. 

At present, most of the available literature is specific to biomarkers for certain cancer types such as biomarkers for breast cancer [31], biomarkers for colorectal cancer [32], biomarkers for prostate cancer [33], biomarkers for ovarian cancer [34], biomarkers for cervical cancer [35], etc. 

In this review, we focused mainly on more recent cancer-detecting methods such as cancer biomarker detection in various cancer types and ways in which this biosensor-mediated biosensing technology can show advantage over traditional detection methods. We also explored various types of cancer biomarkers, availability of traditional cancer biomarkers, recent developments in finding novel cancer biomarkers and their respective detection methods. 

## 2. Cancer Biomarkers 

The conventional diagnostic technologies such as MRI (magnetic resonance imaging), CT (computerized tomography) scan, ultrasound and biopsy were not effective for cancer detection at primary stages; this is because of their dependency on tumorigenic properties or phenotypic characters of a tumor [36,37,38,39,40]. Cancer is a very complex disease, with many epigenetic as well as genetic modifications which might alter the cell signaling process, related to development and resulting in tumorigenic transformation and malignancy [41]. For almost all cancer patients, researchers and clinicians expect tests or methods that might diagnose cancer significantly earlier, provide better prognosis, and that can allow for increased survival rates. Cancer markers have been used over the past few decades in the oncology field. Biomarkers are molecules of biologic emergence found in blood, tissues, various body fluids such as urine, cerebrospinal fluid, or different body tissues that are elevated is the indicative of an abnormal disease or condition with cancer. Human body responsiveness to any therapy can be observed and regulated through biomarkers. These also exist on or in cancer cells. Cancer biomarkers are possibly one of the most potential tools to detect cancer early [42,43,44,45,46].

### 2.1. Clinical Significance of Cancer Biomarkers

**Figure 2:**
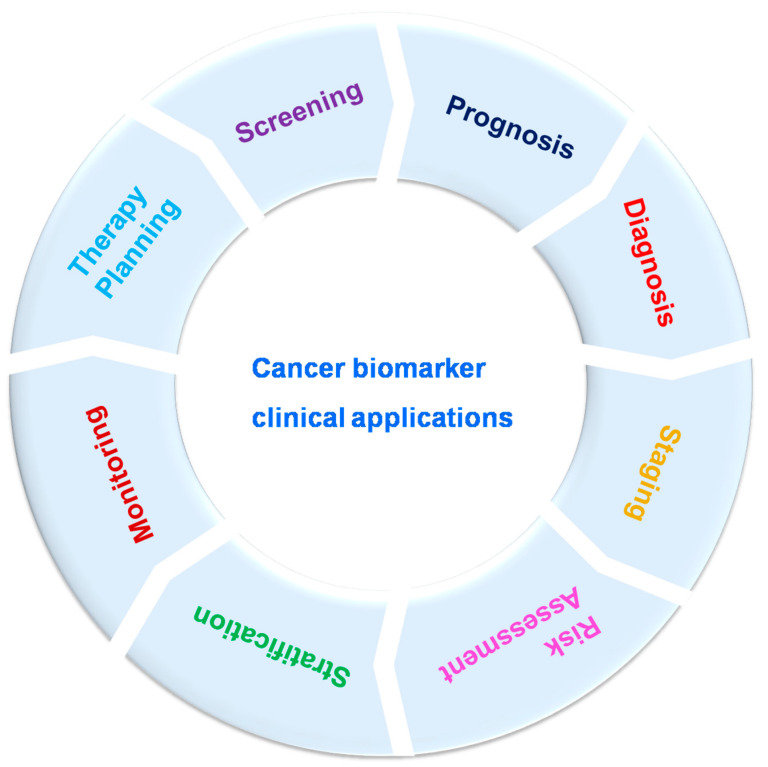
Cancer biomarker clinical applications.

Cancer biomarkers can be utilized for cancer patient evaluation in different clinical levels, as well as disease screening, prognosis, diagnosis, staging, risk assessment, stratification, therapy planning and monitoring (Figure 2). Still, to date, several cancer markers have indicated poor validity and efficacy, especially in the most widespread cancers such as lung and breast cancers. Cancer biomarkers are bio-molecules necessary for remodeling throughout cancer which maintain excessive clinical relevance. These can be enzymes, iso-enzymes, nucleic acids, proteins, metabolites. Biomarkers are classified into three types based on their clinical advantage: prognostic, predictive and diagnostic. Prognostic biomarkers provide details about course of recurrence of the disease; patient response to the treatment is estimated by predictive biomarkers; disease detection can be performed by using diagnostic biomarkers [43,47,48,49]. The difference in the level of any unique biomarker in a cell or tissue is often used as evidence of tumor expansion. Biomarkers also play potential role in differentiating benign and malignant tumors and one type of malignancy from another type; specific biomarkers are helpful in unique settings, other biomarkers can be involved in multiple settings [48]. Biomarkers might be helpful to estimate a person’s chance of developing tumors/cancer. For example, a person having a solid family network (via ancestors) with ovarian cancer might receive a genetic test to decide whether they are acting as a carrier for a specific germ line modification or mutation, such as BRCA1, which could cause potential chance of developing breast and/or ovarian cancer [50,51]. Biomarkers might be helpful to determine malignancy in fit populations. A frequently utilized component for screening is PSA (prostate-specific antigen). It was approved by Food and Drug Administration (FDA) in 1986. Enhanced screening in male population above 50 years old lead to growth in the identification of prostate cancer. These kinds of traditional biomarkers also have limitations. In the previous decade, U.S. Preventive Services Task Force survey noted that an adequate documentation for common diagnosis with PSA [52,53,54]. Biomarkers were used to monitor prognosis and possibility of cancer reappearance irrespective of therapy/treatment. The clinical and pathological properties of a tumor could be useful for the prediction of cancers. Recently, modern techniques were used to evaluate prognosis of independent tumors; for example, a large number of genetic marks that had been matured in breast cancer might be useful to evaluate the identification for an individual patient depending on tumor assessment [55,56]. During breast cancer (metastatic) condition, circulating tumor cells are indicative of overall survival [57,58,59]. Biomarkers could be utilized as stimulus changers or prognostic factors for unique type of therapy, as well as to select the effective type of treatment. KRAS is a predictive biomarker for colorectal cancer; mutations occurring to KRAS in somatic cells are related to low response to anti-EGFR-mediated treatment [60,61]. Likewise, HER2 over-expression in the breast cancer as well as gastric cancer anticipates for stimulus to anti-Her2 drugs such as trastuzumab [62,63,64,65,66,67], and over-expression of estrogen receptor anticipates for stimulus to anti-endocrine therapy or treatment such as tamoxifen in breast cancer [68,69]. Identification of novel cancer biomarkers might help in quick and efficient diagnosis as well as monitoring of cancer progression. 

### 2.2. Identification of Novel Cancer Biomarkers

Possible cancer biomarkers could be recognized through various approaches. An excellent way to recognize novel biomarkers mostly depends on biological nature of tumor and nearby environment of a tumor or metabolism of the drugs or biological products. With most recent studies and new information related to cancers and appearance of latest technology, cancer biomarker detection is performed frequently these days with applying a discovery approach. In this approach, few major areas of research such as gene expression arrays, proteomic technologies (mass spectroscopy, LC-MS/MS, MALDI-MS), and high throughput sequencing can be used to rapidly recognize unique biomarkers or pool of biomarkers which can show difference in the middle of cohorts. Expansion of sophisticated software algorithms for large data analysis has emerged in rapid advancement in the identification of novel cancer biomarkers. Openly available software programs can sort these data and compare the sequences to annotated genome databases to permit quantitative comparative evaluation of proteomes from multiple sources such as tumor area and nearby healthy tissues. Thus, over-expressed or down-regulated proteins in a cancer cell can be identified as putative cancer biomarkers. Huge amount of data produced with these technological methods mean that special attention needs to be directed toward both developing a study plan and conducting a large data analysis. Moreover, it is crucial to reduce the possibility of detecting relationships that are eventually determined to provide false-positive results. The most crucial features of biomarker improvement and identification to consider in depth include mindful study pattern to minimize any kind of bias, extensive evaluation, validation, and accurate communication of the results [48,70,71,72,73,74,75,76,77,78,79]. 

### 2.3. Cancer Biomarkers Currently Used in Clinical Settings

A cancer biomarker is a molecule existing inside and/or generated by tumor cells or surrounding cells in tissues or organs in stimulus to tumor or certain noncancerous (benign) situations, which can provide information about cancer, mostly which stage it is, whether it can respond to treatment, and what type of therapy might be useful. Here, few recent cancer biomarkers used in clinical practice are explained. New cancer biomarkers become available continuously, and they may not be explained below [27,40,44,80,81,82].

#### 2.3.1. AFP (Alpha-Fetoprotein)

AFP (Alpha-fetoprotein) is one of the leading biomarker. Early fetal life of a baby (mostly yolk sac and liver) produces AFP during pregnancy. AFP can be detected in huge amounts in serum of the patients with specific tumors. According to Yuri Semenovich Tatarinov, AFP was first accepted as an antigen unique for human HCC (hepatocellular carcinoma) [83,84]. The scientific literature has explained that AFPs are classified into subtypes based on their dissimilar affinities to LCA (lens culinaris agglutinin), such as AFP-L1 (LCA unreactive AFP), AFP-L2 (LCA mild active AFP) and AFP-L3 (LCA active AFP). In healthy individual serum, an average level of AFP is less than 20 ng 8 mL^−1^ [85,86,87]. AFP is widely established for HCC (hepatocellular carcinoma) diagnosis. Moreover, in congenital tyrosinemia, cirrhosis, hepatitis (alcohol-induced), hepatitis (viral-induced), ataxia-telangiectasia syndrome or in several malignancies such as testicular cancer, liver cancer, gastric cancer and nasopharyngeal cancer elevated AFP levels may also be present. Hence, sensing the AFP values is completely mandatory in clinical settings. High recognition rates of molecular assays have been obtained in quantitative observation of AFP due to their specificity and unalterable affinity of the probes to molecular targets [88,89]. 

#### 2.3.2. PSA (Prostate Specific Antigen)

PSA (prostate-specific antigen) was one of the first recognized cancer biomarkers, utilized to detect and screen prostate cancer in clinical setting. It has been shown that increased levels of PSA can directly relate to prostate cancer. Human regular PSA level is 4 ng/mL. According to the study by Smith et. al, almost 30% of individuals with PSA values higher than normal (range of 4.1–9.9 ng/mL) were diagnosed with prostate cancer [90]. Along with this, raised PSA values may also indicate benign tumors (non-fatal), prostatitis/prostate inflammation or benign prostatic hyperplasia. Therefore, elevated values of PSA may not consistently suggest malignant tumors. There is a fact that caused reasonable controversy about using regular PSA screening to detect prostate cancer. Small-sized tumors identified by PSA screening may grow very slowly; death caused by small tumor might not be feasible in an individual lifetime. Moreover, it is very expensive to treat such slow-growing tumors. Other frequent issue with PSA screening is false-positives. This limitation of PSA testing can be overcome by modern biosensing technology mediated by biosensors [79,84,91,92,93]. PSA detection can be performed by various methods; these traditional methods are time-consuming as well as expensive. Yang et al. explained a graphene oxide/ssDNA-based biosensor integrated with dual antibody-modified PLLA NPs to amplify electrochemical signals for the effective and rapid electrochemical capture of PSA in serum samples from prostate cancer patients. The detection limit for PSA was 1 ng/mL, which achieved a wide linear range of 1–100 ng/mL for PSA. This is one of the examples that shows the usefulness of modern biosensing technology mediated by biosensors [94].

#### 2.3.3. RCAS1 (Receptor-Binding Cancer Antigen)

RCAS1 (receptor-binding cancer antigen) overexpression data has been described in many gastric carcinomas; it is related with progression of gastric cancer. Further, RCAS1 is also suggested as a cancer biomarker for poor prediction in breast, esophageal, endometrial, bladder cancers and is associated with tumor weakening in pharyngeal carcinoma and laryngeal cancer. RCAS overexpression is observed in several types of cancers. Thus, it serves as a potential biomarker for cancer detection and prediction [95,96,97,98]. 

#### 2.3.4. CA 15-3 (Cancer Antigen 15-3)

The most predominant cancer marker for breast cancer identification as well as monitoring is cancer antigen 15-3; additional biomarkers that are related to breast cancer are CA 27.29, BRCA1, BRCA2 and (carcinoembryonic antigen) CEA [44,99,100]. This particular marker is frequently used on a clinical level to monitor the therapy for breast cancer in its advanced stages. During breast cancer, CA 15-3 values increased by 10, 20, 30 and 40 % at various stages first, second, third and fourth stage [49]. Tampellini et. al showed the connection between breast cancer and CA 15-3 levels, also mentioning that before treatment, patients with levels of 30U/mL had notably higher survival rate compared to patients with higher values. Raised CA 15-3 values correspond with extensive metastasis [101,102]. Other research data explained that raised CA 15-3 levels post cancer treatment can be a sign of disease recurrence. Nowadays, to determine breast cancer treatment, protocol CA 15-3 values are considered along with risk factors (negative) such as PR/ER condition as well as iHer-2, cancer stage and tumor dimension. Hepatitis, endometriosis, pelvic inflammatory disease, lactation and pregnancy are conditions other than cancer where CA 15-3 levels are increased [103].

#### 2.3.5. Cancer–Testis (CT) Antigens 

Cancer–testis antigens are a specific type of cancer biomarkers. They are expressed in various cancers. CT antigen expression is limited to male germ cells of the testis but not shown in normal adult cells. These antigens are also expressed in trophoblast and ovary cells. Therefore, CT antigens have been considered as possible immunogenic targets for cancer vaccines (cancer immunotherapies). CT antigen autoantibodies have been studied as potential cancer biomarkers [104]. NY-ESO-1 (NewYorkesophagealsquamouscellcarcinoma1) is encoded by *CTAG-1B*; this is the class of antigens with high immunogenic nature which induces very robust cellular and humoral immune response in NY-ESO-1-positive cancers. Antibody titer to NY-ESO-1 has been shown to relate with disease progression. One of the best benefits of using CT autoantibodies as tumor biomarkers is the fact that they are easy to obtain and are also more stable proteins present in serum compared to tissues obtained via biopsy. Thus, they can be useful for cancer progression and recurrence [105,106,107,108,109]. Major limitation of CT antigens is the fact that many cancers express CT antigens, and they are rarely tumor-specific. Biosensor technology can obtain a profile for CT antigen, which can enhance the use of these antigens in cancer prognosis and diagnosis [110,111,112].

#### 2.3.6. CA 125 

The rise in CA 125 levels is primarily related to ovarian cancer. It is further correlated with several different cancers such as cervix, lungs, breast, liver, pancreas, uterus, stomach and colon cancers. Enhanced levels of CA 125 are also observed in various non-pathological conditions such as menstruation and pregnancy [8,87,113]. A total of 90% of women with advanced stage ovarian cancer and 40% of humans with intra-abdominal malignant tumors also exhibit high CA 125 levels. Still, approximately 50% of patients diagnosed with primary stage ovarian cancer show normal CA 125 levels [87,88,89]. Other germ cell origin biomarkers such as alpha-fetoprotein/AFP, human chorionic gonadotrophin/HCG as well as Lactate dehydrogenase/LDH are also connected to ovarian cancer [114,115]. Increase in CA 125 levels can be used to detect the development of benign tumors into malignant tumors. Enhanced CA 125 levels are also used to identify treatment failure as well as disease recurrence (e.g., high CA 125 levels after bilateral salpingo-oophorectomy or total abdominal hysterectomy, which may occur after first line chemotherapy) [116,117,118,119,120]. Altogether, CA 125 is an extremely essential biomarker for detection of cancer, and also for cancer progression monitoring and treatment. 

#### 2.3.7. CA 19-9

This antigen was first identified in pancreatic and colon cancer patient’s serum in 1981. It is a Lewis antigen of the MUC1 glycoprotein [8,121]. CA 19-9 normal level in serum is less than 37 U/mL. In the recent decade, on a clinical level, CA 19-9 biomarker has become extremely useful for the diagnosis of pancreatic cancer. Normal human serum CA 19-9 levels can play an outstanding role in clinical diagnosis of urothelial and gastric cancers [122,123]. Consequently, there is a necessity to improve highly sensitive methods which can detect CA 19-9 values in patients with cancer.

#### 2.3.8. Nse (Neuron-Specific Enolase)

This neuron-specific enolase is a popular and unique marker for SCLC as well as NSCLC non-small cell lung cancer. It has a crucial role in glycolysis; in 1980s, NSE expression was noted in SCLC cells. From that time, it has been used as potential biomarker for lung cancer, able to detect increased values of NSCLC and acting as crucial predictor for patient survival (it is not dependent on remaining prognostic factors) [124,125,126]. NSE is also a unique marker for neuro-endocrine cells. Raised NSE values in body fluids might be an indication of tumor proliferation and staging determination in some of brain tumors [127,128]. In the recent decade, the value of NSE for prognosis in cancer patients is debatable. Therefore, it is mandatory to improve sensitive techniques to perceive Nse values in patients with cancer.

#### 2.3.9. Tdt (Terminal Deoxynucleotidyl Transferase)

Tdt is an intracellular marker which has detected in the bone marrow as well as blood (mononucleate) cells in leukemia patients during diagnosis. Overall, TdT values are remarkably raised in several lymphocytic lymphomas. Tdt might be helpful to identify specific leukemia type and supportive sign for the solution of therapy [129,130,131,132].

#### 2.3.10. CYFRA21-1 

Cytokeratin-19 fragments/CYFRA21-1 have been extensively studied in patients with NSCLC and are widely utilized as predictive, prognostic markers. They have 56% sensitivity as well as 88% specificity when the value is >1.5 ng/mL. Researchers used maximum cut off value for CYFRA21-1 similar to ≥3 ng/mL; it had shown increased specificity at 97%. It has potential capability in lung, esophagal cancer prediction. Raised values are certain but barely sensitive. These values are strongly connected with cancer metastasis. Recent reports explained that CYFRA21-1 is used as independent prediction factor for various phases of lung cancer. This might function as definitive distinction between benign and malignant lung cancer along with clinical information [133,134,135,136,137]. 

Table 1 demonstrates the summary of various cancer biomarkers available in the literature for the detection of cancer diseases. Significant biomarkers are assembled in the table including their respective cancer diseases and their advantages. 

## 3. Importance of Finding Novel Bio-Sensing Methods to Detect Cancer Biomarkers

Biomarkers might have different molecular origins, such as changes to nucleic acids including RNA, DNA (amplification, translocation, point mutation, loss of heterozygosis) and protein (antibodies, tumor suppressors, oncogenes and hormones). Some of these cancer biomarkers are convincing and extremely crucial for early diagnosis of tumors. Some of the biomarkers need to exhibit adequate specificity and sensitivity for translation into clinical use or for monitoring of disease progression. In this area, biosensing technology can potentially play a crucial role to improve early diagnosis of cancer. The traditional PCR (polymerase chain reaction) or ELISA (enzyme-linked immunosorbent assay) techniques for cancer biomarker identification have some technical limitations, including utilization of costly chemicals and expensive machines in every single assay, which could delay the detection. Moreover, these types of techniques are not capable of constant monitoring in patients throughout treatment. Along with this, multiple pathways are interlinked with cancer cells and these cells express more than one biomarker. Therefore, simultaneous identification of different biomarkers for accurate prognosis and diagnosis is indispensable [8,42,48,138,139,140,141,142,143]. Biosensors offer great potential sensing methodology platforms for the detection of various cancer biomarkers. Specifically electrochemical and optical biosensors based on the affinity, chemical, bio-affinity recognition elements attract great interest. In the following section, we comprehensively discuss the electrochemical and optical sensing strategies for cancer disease diagnosis.

**Table 1:** Biomarkers used in cancer detection.

Tumor/Cancer Biomarker	Type of Cancer/Infected Location	Application	References
AFP	Liver (HCC)	Identifying recurrence, treatment monitoring, disease diagnosis	[144,145,146,147]
PSA	Prostate gland	Screening, identifying recurrence, treatment monitoring, disease diagnosis	[148,149,150]
CA 15-3	Breast	Treatment monitoring	[103,151,152]
CT antigens	Prostate, liver, lung,bladder, skin	Diagnosis, prognosis	[105,106]
CA27.29	Breast	Monitoring	[69,153]
RCAS1	Stomach	Detection, prognosis	[96,97,98]
CA 19-9	Pancreas, colon	Treatment monitoring	[122,154]
CEA (Carcinoembryonic antigen)	Colon, liver	Screening, Identifying recurrence, Treatment monitoring, Disease prognosis	[155,156]
Calcitonin	Thyroid gland	Treatment monitoring, Disease prognosis	[157]
ER & PgR (Estrogen, progesterone receptors)	Breast	Stratification	[158,159,160]
HER2	Lung, breast	Monitoring therapy	[64,161,162,163]
CA 125	Ovary	Prognosis, identifying recurrence, treatment monitoring, disease diagnosis	[88,89]
HCG-β	Ovary, testis	Diagnosis, staging, identifying recurrence, treatment monitoring	[164,165]
Tdt	Blood/leukemia	Diagnosis	[129]
NSe	Lung	Prognosis	[125,166]
Thyroglobulin	Thyroid	Treatment monitoring	[167,168]
PCA3	Prostate gland	Prognosis	[169]
NY-eSO-1	Skin/melanoma	Progression monitoring	[170]
EGFR	Lung	Diagnosis and monitoring therapy	[171,172]
KRAS, ALK	Lung	Diagnosis and monitoring therapy	[173,174]
CD30	Blood/Leukemia	Diagnosis and prognosis	[175,176]
NMP 22	Bladder	Screening, treatment monitoring, disease prognosis	[177,178]
CYFRA21-1	Esophagus	Prognosis, Treatment monitoring	[179,180,181]
BCL2	Blood and breast	Diagnosis, treatment plan	[182,183,184]
BCR-ABL fusion gene	Bone marrow, blood	Prognosis, treatment determination, monitoring	[185,186]
CD20	Blood	Treatment determination	[187]
CD22	Blood	Treatment determination, diagnosis	[188]
CD25	Blood	Treatment determination	[189]
FGFR2 & FGFR3	Bladder	Treatment determination, therapy	[190,191]
Fibrin-fibrinogen	Bladder	Treatment determination, monitoring	[192,193]
SMRP	Leukemia	Progression monitoring	[194,195]
ROS1	Lung	Treatment determination	[196]
OVA1	Ovary	Prognosis	[197]
VMA	Brain	Diagnosis	[198]

## 4. Analytical Diagnostics Methodologies for Cancer Biomarkers Screening

Analytical biosensing methodologies for the detection of various analyte molecules including cancer biomarkers, pharmaceutical drugs, and agricultural toxins are recently rapidly growing. These techniques have several unique advantages such as point-of-care diagnosis, miniaturized portable instrumentation, cost-effectiveness and, moreover, user-friendliness to the end users. In this current section, we discuss some of the recently published works related to the optical- and electrochemical-based biosensors for cancer biomarker detection. 

O. Awatef et al. reported a selective, sensitive, and inexpensive aptamer-based SERS biosensor for detection of prostate-specific antigen in human serum. Here, they have been using 1D (1 Dimensional) Silicon nanowires as a transduction material due to their advantage large surface area. These materials prepared from N-doped Si(100) wafers by metal-assisted chemical etching method. AgNPs were deposited on SiNWs through the electroless deposition technique to enhance the optical signal properties of the sensor. AgNPs/SiNWs were further functionalized with self-assembled layer of hexanethiol by incubating the SERS substrate of MCH to avoid the non-specific binding of the analyte. The developed sensor exhibited a good response in the dynamic range from 0.1 to 20 μg·L^−1^ with a limit of detection of 0.1 μg·L^−1^. Moreover, this sensing platform selectively and sensitively detected PSA in spiked PBS solutions [199].

M. Sachin et al. developed the fabrication of a tailored biofunctionalized interdigitated capacitor electrode (Ti/Pt imprinted) for label-free PSA detection. This sensing platform exhibits rapid detection within 3 s, stability up to many weeks, reusability and reproducibility; it is also requires low volume and easy to operate. Here, interdigitated capacitor (IDC) chip was initially functionalized with Ti/Pt metal by e-beam deposition process. Electrode surface was activated by placing it into piranha solution to formation of hydroxyl groups on the surface. Then, further formation of an amine group on the surface by APTES solution was drop-costed, and glutaraldehyde solution was applied to generate an antibody through cross-linking. Later anti-PSA was fabricated on the electrode surface, and further immobilization of PSA onto the bio surface has performed to verify the interactions through capacitance. APTES and glutaraldehyde increase the positive capacitive response of IDC-based PSA biosensor and are treated as reference value. By using LCR meter, the change in capacitance variables with respect to changing in concentration of target protein has been calculated [200].

Jose Ribeiro et al. developed new biosensing methodology by merging two different techniques, surface plasmon resonance spectroscopy and electrochemical technique for the detection of breast cancer biomarker carbohydrate Antigen 15-3 to monitor disease progression. Two steps are mainly involved in this process: (i) direct SPR monitoring interaction between surface immobilized antibody and CA 15-3 antigen, performed until adsorption reached equilibrium, and (ii) electrochemical measurements at the SPR gold surface and the resulting immunosensor selective detection for the breast cancer biomarker CA 15-3 protein [201]. 

**Figure 3:**
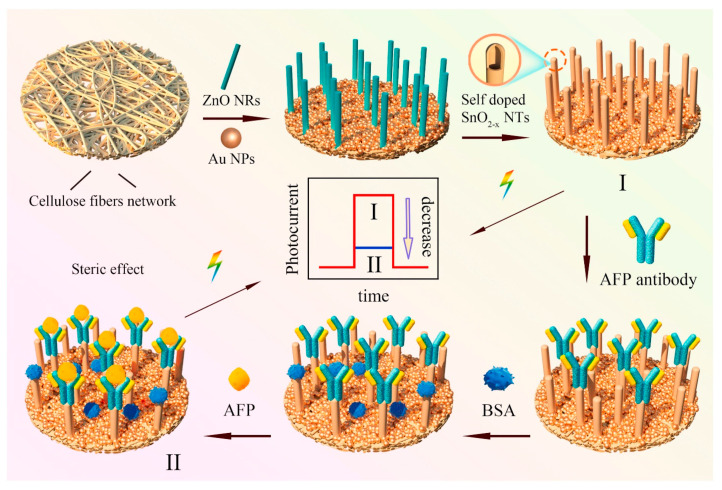
Schematic illustration of the construction of paper-based Sn-doped SnO_2-x_ sample and assay procedures for the specific detection of cancer biomarker alpha fetoprotein. Reproduced with the permission. [202] Copyright 2021, Elsevier.

D. Haihan, et al. performed investigations to explore the construction of paper-based photo electrochemical (PEC) biosensors with 1D self-doping SnO_2_ nanotubes for selectively detection of alpha fetoprotein (AFP). (Figure 3) With the template consumption technique, paper-based 1D-domed SnO_2_ nanotubes have been created from template ZnO nanorods. Additionally, a method of Sn self-doping was suggested to make it easier to separate photo-induced charge carriers and improve the harvesting of visible light. Additionally, self-doping of Sn can reduce the recombination rate of charge carriers and narrow the band gap of SnO_2_ nanotubes, which results in a significant increase in photocurrent intensity under visible light illumination [202]. 

W. Qiong et al. reported the new class of 2D Ti3C2-MXene nanosheet-based SPR biosensor for detection of carcinoembryonic antigen (CEA) cancer biomarker with high specificity and reproducibility. A novel class of ultrathin Ti3C2-MXene possesses hydrophilic biocompatible surface, which can be used as a biosensing material. Ti3C2 -MXene nanosheets were coated with AuNPs using a chemical reduction method and further modified with SPA to improve detection sensitivity or orient purpose. Later immobilization of anti-monoclonal CEA(Ab1) has performed to capture the analyte CEA. Here, Ti3C2-MXene-based SPR sensing platform exhibited significant performance for detection of CEA in real serum samples [203]. 

Table 2 demonstrates the summary of various optical screening methods based on different recognition matrices for the screening of cancer biomarkers. Significant articles with the transduction techniques, bio/chemical recognition matrices, dynamic working calibration range and limit of detection are assembled in the table. 

### Electrochemical Sensing Methodologies

Electrochemical biosensing methodologies for the detection of various analyte molecules including cancer biomarkers, pharmaceutical drugs [218,219,220,221], and agricultural toxins are recently rapidly growing. These techniques have several unique advantages such as point-of-care diagnosis, miniaturized portable instrumentation, cost-effectiveness and, moreover, user-friendliness to the end users. In this current section, we discuss some of the recently published works related to the electrochemical-based biosensors for cancer biomarker detection. 

Huiqing Yang’s team recently developed electrochemical aptasensor for the specific detection of carcinoembryonic antigen (CEA) biomarker. In this investigation, the authors proposed the future of a new antifouling material MXC-Fe_3_O_4_-Ru on functional 2D nanomaterial-modified magnetic gold electrode (MGE). The ferrocene-modified carcinoembryonic antigen aptamer sequences were immobilized on the MGE/modified electrode surface with amido bond chemistry. Electrochemical signal of ferrocene decreases and [Ru(NH3)_6_]_3_ signal fixed on the electrode remains unchanged. The ratio of the electrochemical signals of ferrocene and [Ru(NH3)_6_]_3_ is proportional to the CEA concentration. Even in the complex samples, biosensors can reach high accuracy, selectivity and sensitivity for the detection of targets because of excellent antifouling performance and good conductivity [222]. 

João G. Pacheco et. al. developed an electrochemical sensor based on molecularly imprinted polymer to monitor breast cancer biomarker CA 15-3. In this work, the screen-printed gold electrodes were modified with the MIP recognition matrices. This MIP-based sensor was demonstrated in the electrolytic solution hexacyanoferrate (II/III) as redox probe for measuring the CA protein MIP-binding interactions. Interestingly, the peak current density increases with respect to the CA 15-3 concentration in adynamic range between 5 and 50 U mL^−1^ with the detection limit of 1.5 U mL^−1^. The prepared MIP sensor is low cost and works efficiently for fast (15 min) analysis [223]. 

**Figure 4:**
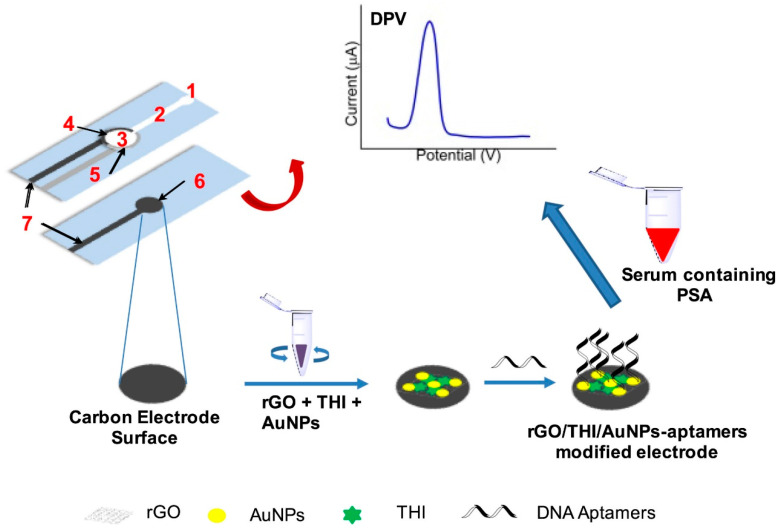
Schematic representation of microfluidic paper-based electrochemical aptasensor for the specific detection of prostate-specific antigen (PSA) in clinical samples. Reproduced with the permission. [224]. Copyright 2018, Elsevier.

Other efforts have focused on the fabrication of label-free electrochemical aptasensor with the integration of microfluidic paper device for the specific detection of prostate specific antigen (PSA) in clinical samples. Screen-printed gold electrodes were fabricated with wax-printed technology. (Figure 4) Au-SPE surface is modified with the reduced graphene oxide and gold–thionine nanoparticle composites. Then, DNA aptamer was immobilized on top of the modified SPE. Afterward, the fabricated aptasensor was tested for the specific detection of PSA for diagnosing prostate cancer disease [224]. 

In another report, an electrochemical aptasensor was proposed by *Leila Farzin’s* research team for the specific detection of CA-125 cancer biomarker. The proposed sensing platform consists of polycrystalline nanofibers coupled with amidoxime-doped silver nanoparticles. The authors reported that hybrid nanomaterial-modified sensor surface helped in better immobilization of aptamer sequences and obtaining sensitive detection limits for CA-125 biomarker detection in ovarian cancer-infected patients [225]. 

Another interesting system that was established was a sandwich-type electrochemical aptasensor to measure carcinoembryonic antigen and cancer antigen 15-3(CA 15-3). The proposed sensing platform consists of a three-dimensional graphene gel embedded with gold nanoparticles (AuNPs/3DGH). This biosensing transducing layer helps in better immobilization of the redox-labelled aptamer sequences. Affinity interaction between aptamers and respective cancer biomarkers (CEA and CA 15-3) was recorded with square wave voltammetry methods by measuring the change in redox probe electroactive signals. This proposed sandwich aptasesing assay exhibited good limit of detection value in nanomolar linear range. The obtained results are comparable with the ELISA method [226]. 

**Figure 5:**
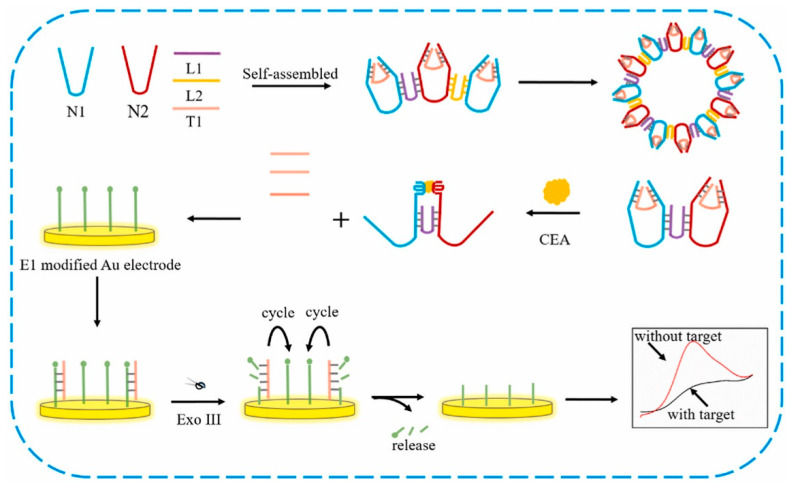
Schematic representation of label-free 3D DNA nanoprobe DNA tweezers-based electrochemical sensor for the detection of carcinoembryonic antigen (CEA) biomarker. Reproduced with the permission [227]. Copyright 2018, Elsevier.

In another report, a DNA nano-tweezer-based electrochemical sensor was developed for sensing and specific detection carcinoembryonic antigen biomarker. (Figure 5) DNA nano-tweezer is a DNA nanomachine used to enhance the sensing performance of electrochemical biosensor. Here, three-dimensional DNA nanomachine possesses the more active sites that could help in enhancement of the competence of reaction. This is the first kind of 3D DNA nanoprobe used in electrochemical sensing platform to obtain stable and reproducible results. Sensor exhibited good electroanalytical performance towards the target biomarker (CEA) with a detection limit of 4.88 fg mL^−1^ [227].

Some other designs have been recently proposed by Navid Taheri et. al. for multiplexed determination of biomarkers alpha-fetoprotein (AFP) and carcinoembryonic antigen (CEA). Here, the sensing assay consisted of an electropolymerized polypyrrole conducting polymer, methyl orange layer and a DMIP layer on FTO surface. The target biomarker and MIP interactions were recorded with the electrochemical impedance spectroscopy by measuring impedance values. The sensor exhibited promising results in the dynamic range of 5–104 and 10–104 pg mL^−1^ and detection limits of 1.6 and 3.3 pg mL^−1^ for CEA and AFP, respectively [228].

**Figure 6:**
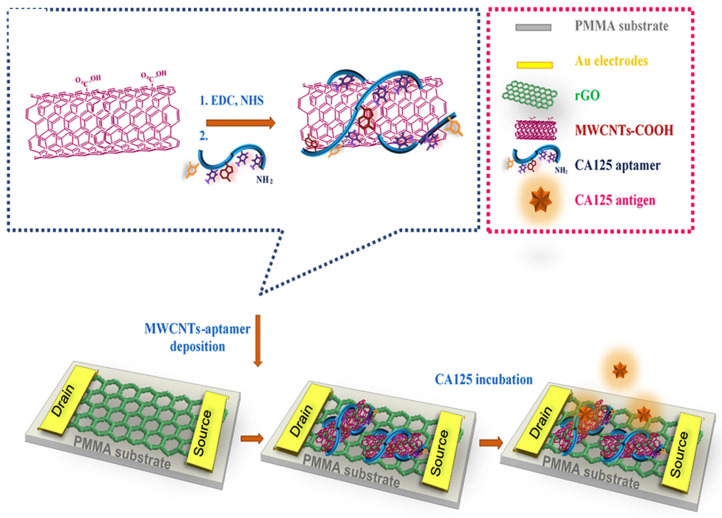
Schematic representation of rGO-FET-based electrochemical aptasensor for the specific detection of CA 125 ssDNA biomarker. Reproduced with the permission [208]. Copyright 2018, Elsevier.

M. Samira et al. developed a novel method for ovarian cancer antigen detection by ultrasensitive flexible aptasensor based on functionalized CNT-reduced graphene oxide nanocomposite. (Figure 6) Reduced graphene oxide film was prepared by modified Hummer’s method. The CA 125 ssDNA aptamer sequences were immobilized on MWCNTs surface by amide bond formation. Then, fabrication of rGO was performed on the polymethyl methacrylate (PMMA) surface by using the polishing method. Here, gold source and drain electrode deposited on graphene film, and the surface of graphene was modified with MWCNTs/CA 125 aptamer through π-π interaction. Overall, this technique specifically and selectively detects the CA 125 biomarker from serum [208].

Table 3 summarizes the recently reported literature related to the electrochemical biosensor for the detection of various cancer biomarkers based on various recognition matrices for the detection of cancer biomarkers. The reports are clearly classified and assembled in the table with respect to their recognition matrices, dynamic working ranges and limits of detection. 

## 5. Conclusions and Future Perspectives

Overall, this review summarizes the importance of cancer biomarker detection, cancer-causing environments, traditional available screening biomarkers for cancer diagnosis, and novel cancer biomarkers and their advantages over traditional biomarkers. Recent developments in analytical diagnostic strategies including electrochemical and optical transduction methods for cancer biomarkers screening are also addressed. A summary of different cancer biomarkers available in the literature for the detection of cancer diseases are displayed in Table 1, including their respective cancer diseases and their advantages. Summaries of optical and electrochemical screening methods based on various recognition matrices for the detection of cancer biomarkers are assembled in their respective tables. 

Cancer biomarker biosensing assay development consists of several critical challenges, including biofluid separation, real sample analysis, sensitivity, multiplex detection and integration of miniaturized instrumentation [243]. Real sample analysis is the particularly significant challenge; we are aiming to detect the target analyte in presence of several protein molecules. To achieve this, we need to integrate the device with the micro/nanofluidic devices and use different coating layers to protect the assay reading from biofouling studies. A recent publication has explained in detailed manner the way in which the fouling occurs, ways to overcome this problem by using different anti-biofouling coatings, as well as the effect of the fouling on electrocatalytic responses [244]. Finally, the device needs to be integrated with the miniaturized instrumentations, and a specific app/software need to be developed to record recognition element and biomarker interactions. The recent review exclusively discussed the integration of biosensing strategies with the electronics devices and wirelessly operated mobile phones for point-of-care diagnostic applications [245]. These sensor integration challenges could be overcome with the help of interdisciplinary approaches, specifically through collaborations between chemistry, electronics, computer, and nanofabrication experts. This review could be help to the early career researchers who are working in the domains of chemistry, biotechnology, nanotechnology, cell biology and biosensors. Overall, the current review provides new insights to the researchers to develop novel biosensing methodologies for the detection of various cancer biomarkers.

## Figures and Tables

**Table 2:** Optical sensing methodologies for cancer biomarker detection.

No.	Biomarker	Recognition	Method	Linear Range	LOD	Ref.
1	AFP	SiO_2_@CQDs/AuNPs/MPBA	ECL	0.001–1000 ng/mL	0.0004 ng/mL	[204]
2	AFP	1D SnO_2_ NTs	PEC	10 pg mL^−1^–200 ng mL^−1^	3.84 pg mL^−1^	[202]
3	AFP	aptamer-MCHA	Fluorescence	0.1 ng mL^−1^–10 mgmL^−1^	0.033 ng. mL ^−1^	[205]
4	CA 19-9	luminol-AgNPs@ZIF-67	ECL	0.0001–10 U/mL	31 μU/mL	[206]
5	CA 19-9	Ni NCs-Ab_2_	Fluorescence	0.001–48 ng mL^−1^	0.00013 ng mL^−1^	[207]
6	CA 125	rGO-based FET-type aptasensor	Fluorescence	1.0 × 10^−9^-1.0 U/mL	5.0 × 10^−10^ U/mL	[208]
7	CA 125	CA 125/MUC16	SPRI	2.2–150 U/ml	-	[209]
8	CEA	Ti_3_C_2_-MXene/AuNPs/SPA	SPR	2 × 10^−16^–2 × 10^−8^ M	0.07 fM	[203]
9	CEA	HCR and G-quadruplex DNAzyme	Fluorescence	0.25–1.5 nM	0.2 nM	[210]
10	HER2	nanoparticle coated QCM	QCM	10–500 cells/mL	10 cells/mL	[211]
11	HER2	PtAmi	Fluorescence	-	-	[212]
12	HER2	3D DNA walker	Fluorescence	0.5–5 ng mL^−1^	0.01 ng mL^−1^	[213]
13	PSA	NaYF_4_:Yb^3+^, Er^3+^ UCNPs and NaYF_4_:Yb^3+^, Er^3+^@NaYF_4_:Yb^3+^ UCNPs	Fluorescence	0.1 ng/mL–10 ng/mL	0.01 ng/mL	[214]
14	PSA	anti-PSA/MCH/ AgNPs/SiNWs	SERS	0.1–20 μg·L^−1^	0.1 μg·L^−1^	[199]
15	PSA	IDC	FET	0.1−10.0 μL/mL	-	[200]
16	CA-15-3	PHMPF	Fluorescence	2.56 × 10^−5^–1.28 U mL^−1^	2.56 × 10^−5^ U mL^−1^	[215]
17	CA-15-3	SPR gold substrates	SPR	-	0.0998 U mL^−1^	[201]
18	NMP 22	orange emitting quantum dot CdTe/CdS	Fluorescence	2–22 pg mL^−1^	0.05 pg mL^−1^	[216]
19	NMP 22	NCDs	Fluorescence	1.3–16.3 ng/mL	0.047 ng/mL	[217]

AFP—alpha-fetoprotein; ECL—electrochemiluminescence; PEC—photoelectrochemical; SPRI—Surface Plasmon Resonance Imaging; QCM—quartz crystal microbalance; PtAmi—red-emitting exchanged Pt nanoclusters; UCNPs—upconversion nanoparticles; IDC—interdigitated capacitor; PHMPF—prismatic hollow Metal-polydopamine frameworks; NCDs-—nitrogen-doped carbon dots.

**Table 3:** Electrochemical sensing methodologies for cancer biomarker detection.

No.	Biomarker	Recognition	Method	Linear Range	LOD	Ref.
1	AFP	PtNPs/GO-COOH	SWV	3.0–30 ng mL^−1^	1.22 ng mL^−1^	[229]
2	AFP	FTO/PPy-MO DMIP	EIS	10–10^4^ pg mL^−1^	3.3 pg mL^−1^	[228]
3	CA 19-9	Au-SPE/TH	DPV	0.010–10 U/mL	-	[230]
4	CA 19-9	1DMoS_2_ nanorods/LiNb_3_O_8_ and AuNPs@POM	DPV	0.1–10.0 µU mL^− 1^	0.030 µU mL^− 1^	[231]
5	CA 125	MIP@AuSPE	SWV	0.01 and 500 U/mL	0.1 U/mL	[232]
6	CA 125	ITO/Ag NPs–PAN-oxime NFs/aptamer/c-DNA–MB	DPV	0.01 to 350 UmL^−1^	0.0042 UmL^−1^	[225]
7	CA 125	Tb-MOF-on- Fe-MOF	EIS	1 × 10^2^−1 × 10^5^ cell·mL^−1^	19 cell·mL^−1^	[233]
8	CEA	MXC-Fe_3_O_4_-Ru	DPV	1 pg/mL–1 μg/mL	0.62 pg/mL	[222]
9	CEA	IEC-BA	DPV	-	4.88 fg mL^−1^	[227]
10	HER2	SPCE-MWCNT/AuNP	LSV	7.5 and 50 ng/mL	0.16 ng/mL	[234]
11	HER2	polycytosine DNA (dC_20_)	SWV	0.001−1 ng/mL	0.5 pg/mL	[235]
12	HER2	GCE/PEDOT/Gel/Ab/HER2	DPV	0.1 ng mL^−1^–1.0 μg mL^−1^	45 pg mL^−1^	[236]
13	HER2	MIP-AuSPE	DPV	10–70 ng/mL	1.6 ng/L	[237]
14	PSA	AuNPs/rGO/THI-aptamer	DPV	0.05 to 200 ng mL^−1^	10 pg mL^−1^	[224]
15	PSA	aptamer PSAG-1	EIS	0.64–62.5 ng/mL	-	[238]
16	CA-15-3	AuNPs/3DGH	DPV	5.0 × 10^–2^–100.0 U mL^–1^	-	[226]
17	CA-15-3	MIP/Au-SPE	DPV	5–50 U mL^−1^	1.5 U mL^−1^	[223]
18	CA-15-3	CysA/Au NSs/GQDs	SWV	0.16–125 U/mL	0.11 U/ml	[239]
19	NMP 22	Cu-MOFs@SiO2@AgNPs	DPV	0.1 pg∙mL^−1^–1000 ng∙mL^−1^	33.33 fg∙mL^−1^	[240]
20	NMP 22	AuNPs-PtNPs-MOFs	DPV	0.005 ng·mL^−1^ –20 ng·mL^−1^	1.7 pg·mL^−1^	[241]
21	NMP 22	Co-MOFs/CuAu NWs	Amperometric	0.1 pg mL^−1^–1 ng mL^−1^	33 fg mL^−1^	[242]

DMIP—dual-template molecularly imprinted polymer; TH—thionine; IEC-BA—ingenious electrochemical aptamer biosensor; PSA—prostate-specific antigen; AuNPs/3DGH—gold nanoparticle three-dimensional graphene hydrogel.

## Data Availability

Not applicable.

## Abbreviations

AFP	Alpha-fetoprotein
PSA	Prostate-specific antigen
RCAS1	Receptor-binding cancer antigen
CA 15-3	Cancer antigen 15-3
CT antigen	Cancer–testis antigen
CA 125	Cancer antigen 125
CA 19-9	Cancer antigen 19-9
Nse	Neuron-specific enolase
Tdt	Terminal deoxynucleotidyl transferase
CYFRA21-1	Cytokeratin-19 fragments
Carcinoma	Epithelial cell cancer
Sarcoma	Connective tissue/bone cancer
Lymphoma	Lymphatic system cancer
Myeloma	Plasma cell cancer
Leukemia	Blood cancer
BRCA1	Breast cancer gene 1
BRCA2	Breast cancer gene 2
